# Brain computer tomography in critically ill patients - a prospective cohort study

**DOI:** 10.1186/1471-2342-12-34

**Published:** 2012-12-12

**Authors:** Ilse M Purmer, Erik P van Iperen, Ludo F M Beenen, Michael J Kuiper, Jan M Binnekade, Peter W Vandertop, Marcus J Schultz, Janneke Horn

**Affiliations:** 1 Department of Intensive Care Medicine, Academic Medical Center, PObox 22660, 1100, Amsterdam, DD, The Netherlands; 2 Department of Radiology, Academic Medical Center, PObox 22660, 1100, Amsterdam, DD, The Netherlands; 3 Department of Intensive Care, Medicine, Medical Center Leeuwarden, PObox 888, 8901, Leeuwarden, BR, The Netherlands; 4 Neurosurgical Center Amsterdam, Academic Medical Center, PObox 22660, 1100, Amsterdam, DD, The Netherlands

**Keywords:** Computer tomography, Critically ill, Brain imaging, Diagnostic value

## Abstract

**Background:**

Brain computer tomography (brain CT) is an important imaging tool in patients with intracranial disorders. In ICU patients, a brain CT implies an intrahospital transport which has inherent risks. The proceeds and consequences of a brain CT in a critically ill patient should outweigh these risks. The aim of this study was to critically evaluate the diagnostic and therapeutic yield of brain CT in ICU patients.

**Methods:**

In a prospective observational study data were collected during one year on the reasons to request a brain CT, expected abnormalities, abnormalities found by the radiologist and consequences for treatment. An “expected abnormality” was any finding that had been predicted by the physician requesting the brain CT. A brain CT was “diagnostically positive”, if the abnormality found was new or if an already known abnormality was increased. It was “diagnostically negative” if an already known abnormality was unchanged or if an expected abnormality was not found. The treatment consequences of the brain CT, were registered as “treatment as planned”, “treatment changed, not as planned”, “treatment unchanged”.

**Results:**

Data of 225 brain CT in 175 patients were analyzed. In 115 (51%) brain CT the abnormalities found were new or increased known abnormalities. 115 (51%) brain CT were found to be diagnostically positive. In the medical group 29 (39%) of brain CT were positive, in the surgical group 86 (57%), *p* 0.01. After a positive brain CT, in which the expected abnormalities were found, treatment was changed as planned in 33%, and in 19% treatment was changed otherwise than planned.

**Conclusions:**

The results of this study show that the diagnostic and therapeutic yield of brain CT in critically ill patients is moderate. The development of guidelines regarding the decision rules for performing a brain CT in ICU patients is needed.

## Background

Brain computer tomography (brain CT) is an important imaging tool in patients with suspected or proven intracranial disorders. Reasons to perform a brain CT in patients admitted to the intensive care unit (ICU) are failure to wake up after wearing off of sedative medication, neurological deterioration, follow up of known intracranial pathology or evaluation of a neurosurgical intervention. In the fast majority of ICUs, a brain CT implies an intrahospital transport of which the inherent risks are well known [1-4]. The proceeds and consequences of a brain CT in a critically ill patient should outweigh these risks. Therefore, the request for a brain CT in an ICU-patient with minor changes in the neurological condition or in a patient who is doing well clinically after surgery, frequently leads to a debate about the importance of that brain CT in terms of expectations and treatment consequences.

We prospectively collected all brain CT requests, the brain CT results and resulting changes in treatment during one year in two hospitals in order to determine the diagnostic and therapeutic impact of a brain CT in ICU patients. The aim of this study was to critically evaluate the diagnostic and therapeutic yield of brain CT in ICU patients.

## Methods

### Study design

This prospective observational study was performed in two ICUs in the Netherlands - one tertiary 16-bed mixed medical-surgical ICU, and one 30-bed university mixed medical-surgical ICU including neurosurgery. From May 2007 until June 2008 all consecutive brain CTs were included. The study was approved by the local medical ethics committees of the Academic Medical Center (Amsterdam, the Netherlands) and Medical Center Leeuwarden (Leeuwarden, the Netherlands) and was conducted in concordance with the principles of the declaration of Helsinki and good clinical practice. Informed consent from patients or relatives was not deemed necessary by the medical ethical committees given the observational nature of the study.

The decision to perform a brain CT was at the discretion of the attending neurologist, neurosurgeon or intensivist. The physician requesting the brain CT completed a standardized radiological request form, specifying the indication for the brain CT, the expectations regarding results and possible treatment consequences. Options that could be chosen from as expected abnormalities were “hydrocephalus”, “ischaemia”, “vascular occlusion or dissection”, “intracranial hematoma”, “edema (diffuse or local)”, “midline shift”, “herniation” or “other”. More than one expected abnormality could be ticked. Possible treatment options were “insertion of intracranial pressure (ICP) monitor”, “placement of ventricular catheter”, “lumbar puncture”, “craniectomy/craniotomy”, “hematoma removal”, “mannitol infusion ”, “observation”, or “other”.

All brain CTs were evaluated by an independent radiologist. All abnormalities reported by the radiologist, irrespective whether they were old or new, were noted. In case of old abnormalities it was reported if they were increased, unchanged or decreased. The actually applied treatment on the ICU was evaluated by the members of the research team. Possible consequences were “treatment as planned”, “treatment changed, not as planned”, “treatment unchanged”.

Patients were divided in two groups: surgical and medical. Surgical patients were admitted with a subarachnoid or intracerebral hemorrhage, traumatic brain injury, or after neurosurgical intervention. Medical patients were admitted to the ICU for a non- neurosurgical reason.

*Other data collected*: age, gender, ICU admission type (acute or elective), specialism (medical (including neurology), surgical (including neurosurgery)), APACHE II-score, neurological/neurosurgical diagnosis, and, if available, ICP data. Ventilation and administration of vasopressive medication during transport was recorded.

### Definitions

An “expected abnormality” was any finding (or worsening of a previously noted finding) that had been predicted by the physician requesting the brain CT. A brain CT was “diagnostically positive”, if the abnormality found was new or if an already known abnormality was increased. It was “diagnostically negative” if an already known abnormality was unchanged or if an expected abnormality was not found. A brain CT with more than one expected abnormality could only be “diagnostically negative” if all expectations were unconfirmed. The treatment consequences of the brain CT, which were collected from the medical files, were registered as “treatment as planned”, “treatment changed, not as planned”, “treatment unchanged”. To determine the positive predictive value of a diagnostically positive brain CT on patient’s treatment, “treatment as planned” and “treatment changed, not as planned”, were considered as “treatment changed” versus “treatment unchanged”.

### Statistical analysis

Descriptive statistics were used to characterize the study cohort. The chance that a brain CT was followed by a change of treatment policy was expressed as relative risk (RR). Statistical uncertainty was expressed using 95% confidence intervals (CI). A statistical software package (Statistical Package for the Social Sciences, version 16; SPSS Inc., Chicago, IL) was used for the statistical analyses.

## Results

**Figure 1:**
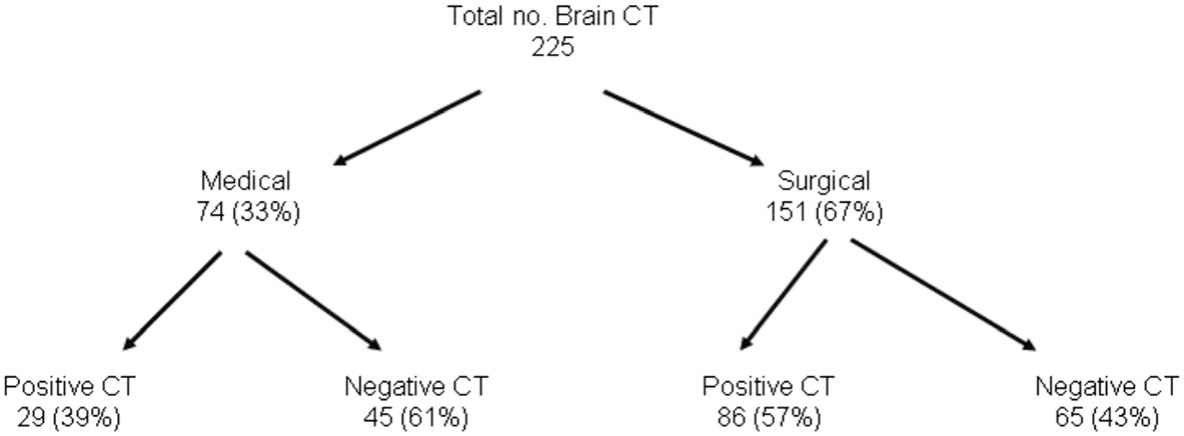
Distribution of brain CTs.

Data of 225 brain CT obtained in 175 patients were collected and analyzed (see Figure 1). The majority of brain CT (207) were made in the university ICU, 18 in the tertiary mixed medical - surgical ICU. Patient characteristics are shown in Table 1. Seventy-four brain CT were performed in 72 medical patients, 151 in 103 surgical patients. In some patients more than one brain CT was performed (23 patients had two, 7 patients three brain CT and 3 patients had four, five and eight respectively). During brain CT transport mechanical ventilation was needed in 188 (83%) transports, vasopressive medication was administered during 80 (35%) transports.

**Table 1:** Patient characteristics, n = 175

**Treatment group**	**n (%)**
Surgical	103 (58)
Medical	72 (42)
Age	Mean (Sd)
All patients	57.3 (15.5)
Surgical patients	53.1 (15.9)
Medical patients	63.2 (12.9)
Gender	n (%)
Male	111 (63)
Reason for admission ICU	n (%)
SAH	30 (17)
Intracerebral hematoma	20 (11)
TBI	44 (25)
Cerebral infarction	4 (2)
Post CPR	14 (8)
Sepsis / pneumonia	17 (10)
Cardiopulmonary surgery	13 (7)
Other	33 (19)
Apache II score	Mean (Sd)
All patients	23.6 (8.2)
Surgical patients	23.6 (7.7)
Medical patients	27.5 (8.9)

Abbreviations: *Sd*, standard deviation, *ICU*, intensive care unit, *SAH*, subarachnoid haemorrhage, *TBI*, traumatic brain injury, *CPR*, cardiopulmonary resuscitation.

Overall, 115 (51%) brain CT were found to be diagnostically positive, i.e. the abnormalities reported by the radiologist were new or increased known abnormalities. In the medical brain CT, 29 (39%) were positive, in the surgical group 86 (57%), *p* 0.01. Tables 2 and 3 displays the abnormalities as expected by the requesting physicians and the abnormalities as reported by the radiologist. All abnormalities, irrespective whether they are old or new, are shown. In most brain CT more than one abnormality was expected. In the medical group a brain CT was often made to exclude an intracranial hemorrhage, this was expected in 36 brain CT, but found in 2 (5%). In surgical patients more brain CT (38%) than expected (23%) showed midline shift. Medical brain CT showed no abnormalities in 45%, in surgical brain CT no abnormalities were reported in 4%.

**Table 2:** Expected radiological abnormalities and results as reported by the radiologist in medical brain CT (n = 74)

**Expected abnormalities**	**n (%)**	**Results radiologist**	**n (%)**
Hematoma	36 (49)	Hematoma	2 (3)
Ischemia / infarction	49 (66)	Ischemia / infarction	34 (46)
Oedema	15 (20)	Oedema	5 (7)
Midline shift	1 (1)	Midline shift	2 (3)
Hydrocephalus	4 (5)	Hydrocephalus	7 (9)
Cerebral abscess	5 (7)	Cerebral abscess	0
No abnormalities	0	No abnormalities	33 (45)
Other	5 (7)	Other	5 (7)

**Table 3:** Expected radiological abnormalities and results as reported by the radiologist in surgical brain CT (n = 151)

**Expected abnormalities**	**n (%)**	**Results radiologist**	**n (%)**
Hematoma	97 (64)	Hematoma	105 (70)
Ischemia / infarction	33 (22)	Ischemia / infarction	33 (22)
Oedema	45 (30)	Oedema	55 (36)
Midline shift	35 (23)	Midline shift	57 (38)
Hydrocephalus	60 (40)	Hydrocephalus	58 (38)
(increase of) TBI	0	(increase of) TBI	0
Cerebral abscess	4 (3)	Cerebral abscess	0
No abnormalities	0	No abnormalities	4 (3)
Other	29 (19)	Other	53 (35)

Table 4 shows the treatment strategies considered when requesting the brain CT. In medical patients, limitation or withdrawal of treatment as possible treatment strategy was mentioned in 16%, in surgical patients in 1%. A large number of surgical patients were treated with an external ventricular drainage system, and brain CT were requested to be informed about the position and effect of the drainage system.

**Table 4:** Considered treatments as indicated on brain CT request form

**Medical brain CTs (74)**	**N, (%)**	**Surgical brain CTs (151)**	**N, (%)**
Craniotomy	9 (12)	Craniotomy	65 (43)
CSF drainage	9 (12)	CSF drainage / reposition EVD	55 (36)
Change medication	16 (22)	Change medication	16 (11)
Continue current therapy	20 (27)	Continue current therapy	36 (24)
Withdraw / limit treatment	12 (16)	Withdraw / limit treatment	1 (0)
Other	4 (0.1)	Other	15 (10)

After a positive brain CT, in which the expected abnormalities were found, treatment was changed as planned in 33%, and in 19% treatment was changed otherwise than planned (Table 5). In 73% of the negative brain CT (in which the expected abnormalities were not found) treatment remained unchanged. Despite such a negative brain CT the treatment was changed as was planned in 16%. The positive predictive value, i.e. the chance that a positive CT scan led to a change in treatment was 0.52 (95% CI 0.42 - 0.61), the negative predictive value, i.e. that chance that treatment was not changed after a negative CT scan was 0.73 (95% CI 0.64 - 0.80).

**Table 5:** Treatment after positive or negative brain CT

**All brain CTs**	**Positive CT, n = 115 (%)**	**Negative CT, n = 110 (%)**
Treatment as planned	38 (33)	18 (16)
Treatment changed, not as planned	22 (19)	12 (11)
Treatment unchanged	55 (48)	80 (73)
CTs in surgical patients	Positive CT, n = 86 (%)	Negative CT, n = 65 (%)
Treatment as planned	29 (34)	11 (17)
Treatment changed, not as planned	19 (22)	8 (12)
Treatment unchanged	28 (44)	46 (71)
CTs in medical patients	Positive CT, n = 29 (%)	Negative CT, n = 45 (%)
Treatment as planned	9 (31)	7 (16)
Treatment changed, not as planned	3 (10)	4 (9)
Treatment unchanged	17 (57)	34 (76)

## Discussion

This is the largest cohort study sofar, investigating the results and consequences of brain CT in patients admitted to an ICU. The diagnostic value of brain CT is low, in only half of the brain CT the abnormalities as expected by the requesting physicians were found. In a brain CT performed in medical patients this is the case in less than fifty percent. The therapeutical consequences of the brain CT are also low, treatment was changed in little more than half of the brain CT.

From the fact that in this study only half of all brain CT showed the abnormalities that were expected, one could conclude that it is difficult for physicians who have performed clinical neurological assessment, to foretell what the brain CT will yield. It is known that neurological assessment of critically ill patients can be hampered due to a combination of the underlying disease, metabolic derangements and (sedative) medication administered. The reason to request a brain CT was different in medical patients, where it was often performed to exclude severe intracranial pathology, such as an intracerebral hemorrhage. Our results showed that an intracerebral hemorrhage is only found in 3% of medical critically ill patients.

Similar results in medical patients were reported by Rafanan et al. and Salerno et al [5,6]. Rafanan reviewed the results of 297 brain CT scans and describes a percentage of 37% of these scans to show acute intracranial abnormalities [5]. Ischemic stroke was found most frequently (49%), which is comparable to the percentage of infarction or ischemia in our study (46%). Salerno et al. reported data of a retrospective study in 123 medical ICU patients in whom a brain CT was performed [6]. In 26 patients (21%) a new finding was described by the radiologist, most often an ischemic cerebral infarct (13). Both studies reported that no patient characteristics or clinical variables could, with certainty, identify patients with either a positive or a negative brain CT. This interesting topic was not addressed in our study.

Balachandran et al. also studied the results of brain CT performed in ICU patients who did not wake up after discontinuation of sedative drugs [7]. In 42 patients, only one patient (2%) with abnormalities explaining the persistent coma was identified.

In surgical ICU patients results and consequences of brain CT have not been published and therefore we were unable to compare our data regarding the diagnostic or therapeutic yield of brain CT in this subgroup. There has been discussion about the yield of repeated brain CT in patients with blunt traumatic brain injury (TBI). Kaups et al. reported that repeated brain CT was unnecessary in patients who did not show deterioration in mental status, elevation of ICP, hypotension or coagulopathy, as the results of the CT did not alter patient management in this group [8]. His results were contradicted by Bee et al., who concluded that in patients with mild TBI, repeated brain CT can identify increase of intracranial lesions even if the patient remains clinically stable [9]. In a systematic review including 30 mostly retrospective studies about the utility of repeated brain CT after blunt TBI, Wang et al. found that progression of injury found on the repeat CT was reported in 8-67% of patients [10]. The number of patients reported to need neurosurgical interventions after the repeated brain CT was 0-54%. Especially patients with severe TBI, presenting with a Glasgow Coma Score of ≤ 8, were at risk of progression of injury necessitating neurosurgical interventions. Despite the large number of manuscripts and therefore patients included in the review, the authors could not determine which subgroup of TBI patients would benefit from repeated brain CT.

Although the number of identified abnormalities in this study is low, it is higher than figures reported from emergency room brain CT results in patients admitted with mild TBI. Several large prospective cohort studies in this population found intracranial traumatic lesions in 6-11% of patients [11-13]. Based on these experiences, prediction rules have been formulated to allow emergency room physicians to work as efficient as possible, without exposing patients to the risk of missing important abnormalities. Given the risks of transports in critically ill patients, similar studies and guidelines would be useful for brain CT in (neuro)surgical and medical critically ill patients. The development of a portable CT scan diminishes the needs for intrahospital transport which can be of great value [14]. Nevertheless, every brain CT leads to radiation exposure, and therefore clinicians should still consider the diagnostic yield of a brain CT and the consequences it will have for the treatment strategy [15].

Some limitations of this study should be discussed. First, in this study we did not collect data on the clinical condition of the patients at the moment the brain CT was requested. Collection of clinical data would have allowed us to correlate the clinical findings to the expectations of the requesting physician and the final results of the brain CT. By doing this, we might have been able to identify clinical warning signs indicative of serious intracranial problems. A future study should address this issue.

Also, the severity of the identified abnormalities on the brain CT and the possible consequences for patients if they had been missed were not recorded. Especially the clinical condition of the patient in the ICU can lead to situations necessitating brain CT, because neurological tests can not be performed, for example when sedative drugs are administered and can not be interrupted. In these patients a brain CT with no new abnormalities can be expected more often than in patients who deteriorate neurologically. The consequences of missed abnormalities on a brain CT would certainly be interesting to study, but this was not included in this project.

## Conclusion

The results of this study show that the diagnostic and therapeutic yield of brain CT in critically ill patients is moderate. However, consequences of missing serious intracranial abnormalities were not addressed in this study. Given the fact that intrahospital transport has risks in critically ill patients, further research is needed to enable the development of guidelines regarding the decision rules for performing a brain CT in ICU patients.

## Abbreviations

APACHE: Acute physiology and chronic health evaluation;CI: Confidence intervals;CPR: Cardiopulmonary resuscitation;CT: Computer tomography;ICP: Intracranial pressure;ICU: Intensive care unit;RR: Relative risk;SAH: Subarachnoid haemorrhage;Sd: Standard deviation;TBI: Traumatic brain injury

## Competing interests

The authors declare that they have no competing interests regarding this study

## Authors’ contributions

IP contributed to data collection, analyses and interpretation of the data and drafting and finalizing of the manuscript. EvI contributed to data collection (e.g. building of database), interpretation of the data, drafting and finalizing of the manuscript. LB contributed to data collection (e.g. reporting of CTs), drafting and finalizing of the manuscript. MK contributed to data collection, drafting and finalizing of the manuscript. JB contributed to analyses and interpretation of the data, drafting and finalizing of the manuscript. PV contributed to study design, data collection and drafting and finalizing of the manuscript. MS has made considerable contributions to conception and design of the study and helped finalization of the manuscript. JH contributed to conception and design of the study, collected data, interpreted data and drafted and finalized the manuscript. All authors have given final approval of the version to be published.

## Pre-publication history

The pre-publication history for this paper can be accessed here:

http://www.biomedcentral.com/1471-2342/12/34/prepub
